# Association Between Insulin Resistance Marker Estimated Glucose Disposal Rate and Cardiovascular Risk in Obesity: Insights From the National Health and Nutrition Examination Survey 1999 to 2018

**DOI:** 10.14740/cr2136

**Published:** 2026-01-04

**Authors:** Xu Hua, Hai Nan Yang, Yao Guo Han, Ming Lei

**Affiliations:** a Department of Critical Care Medicine, Seventh People’s Hospital, Shanghai University of Traditional Chinese Medicine, Pudong New District, Shanghai 200137, China

**Keywords:** Obesity, eGDR, NHANES, CVD, Insulin resistance

## Abstract

**Background:**

This study evaluated the effectiveness of the estimated glucose disposal rate (eGDR), an indicator of insulin resistance, as a screening tool for cardiovascular disease (CVD) in individuals with obesity.

**Methods:**

A cross-sectional analysis was conducted using data from the US National Health and Nutrition Examination Survey (NHANES) covering the years 1999 to 2018. The study included 20,521 participants with a waist-to-height ratio (WHtR) of 0.6 or higher, indicating obesity. Participants were divided into quartiles based on their eGDR levels: Q1 (> 8 mg/kg/min), Q2 (6 - 8 mg/kg/min), Q3 (4 - 6 mg/kg/min), and Q4 (≤ 4 mg/kg/min). Multivariable logistic regression models, adjusted for various demographic, lifestyle, and metabolic confounders, were used to analyze the relationship between eGDR and CVD. The predictive capability of eGDR was assessed using the area under the receiver operating characteristic curve (AUC), restricted cubic splines (RCS) for capturing non-linear relationships, and stratified subgroup analyses.

**Results:**

CVD prevalence significantly increased with decreasing eGDR levels (Q1: 5.3% vs. Q4: 26.2%). After full adjustment for covariates, multivariable regression confirmed that the lowest eGDR quartile (Q4) was strongly and independently associated with a substantially elevated risk of CVD compared to the highest quartile (adjusted odds ratio (OR) = 6.3; 95% confidence interval (CI): 5.53 - 7.17; P < 0.001). eGDR also demonstrated good predictive performance for specific CVD subtypes, with the highest AUC for heart failure (0.715, 95% CI: 0.699 - 0.730). RCS analysis validated a significant non-linear, inverse dose-response relationship between eGDR and overall CVD risk. Subgroup analyses, stratified by age, sex, and glycemic status, consistently demonstrated a significant association between low eGDR and increased CVD risk across all categories (P < 0.001).

**Conclusions:**

Lower eGDR independently and strongly indicated a heightened risk of CVD in individuals with obesity.

## Introduction

Obesity is a significant risk factor for cardiovascular disease (CVD), which is the leading cause of death among those with obesity [1]. Abdominal fat accumulation is closely linked to cardiovascular risk factors, including insulin resistance (IR), dyslipidemia, and hypertension [2].

IR, which involves a decreased response to insulin and causes disruptions in glucose and lipid metabolism [3], has been demonstrated in previous studies as a central mechanistic link in CVD development among patients with obesity. Impaired insulin sensitivity is prevalent in populations affected by obesity, and such metabolic disturbances promote CVD through multiple pathways: IR can induce hyperinsulinemia, impair vascular endothelial function, and drive abnormalities in glucose and lipid metabolism [4, 5]. The estimated glucose disposal rate (eGDR), calculated by integrating glycated hemoglobin (HbA1c), blood pressure, and waist circumference, provides a convenient method for assessing IR, with lower eGDR levels indicating worsening IR [6]. A recent study demonstrated that among individuals with diabetes and prediabetes, eGDR exhibited superior discriminative power for assessing CVD associations compared to other surrogate IR indices [7]. However, the role of eGDR for CVD screening specifically in populations with obesity remains unclear.

This study aimed to evaluate the screening efficacy of eGDR for CVD in population affected by obesity. This study utilized a cross-sectional design and National Health and Nutrition Examination Survey (NHANES) data to investigate the relationship between eGDR and CVD prevalence in adults with obesity, defined by a waist-to-height ratio (WHtR) of 0.6 or higher.

## Materials and Methods

### Study population

The Centers for Disease Control and Prevention (CDC)’s NHANES offers crucial health statistics for shaping public health policy and research. The survey protocol was approved by the Research Ethics Review Board at the National Center for Health Statistics (NCHS). Public access to the datasets employed and reviewed in this study is available on the NHANES website. The data utilized were deidentified; therefore, the study did not need the Institutional Review Board (IRB) approval. An ethics statement was not applicable for this study. Data from NHANES between 1999 and 2018 were used for analysis, excluding participants younger than 20, those missing WHtR or eGDR data, and those with incomplete CVD diagnosis information.

### Definitions of obesity, eGDR, and CVD

Obesity was determined by the ratio of an individual’s waist circumference to their height, both measured in cm. WHtR was chosen as the primary indicator of obesity for this study because it is a simple, effective measure of central adiposity that correlates well with cardiometabolic risk and is less dependent on height compared to body mass index (BMI), making it a robust metric across different populations. A WHtR value of 0.6 or higher indicates obesity.

eGDR (mg/kg/min) was determined using the formula: 21.158 - (0.09 × waist circumference in cm) - (3.407 × hypertension status) - (0.551 × HbA1c in %).

Hypertension status was defined as a binary variable (yes = 1, no = 0) based on a composite of any of the following: self-reported physician diagnosis, current use of antihypertensive medication, or measured average systolic blood pressure ≥ 140 mm Hg or diastolic blood pressure ≥ 90 mm Hg.

CVD diagnosis was established using participant responses from the standardized medical conditions questionnaire in the NHANES (congestive heart failure, coronary heart disease, angina, myocardial infarction, or stroke). Coronary artery disease (CAD) includes conditions such as coronary heart disease, angina, and myocardial infarction.

### Collection of clinical data and grouping

The analysis included a wide range of covariates to account for potential confounders. The variables comprised biological sex, chronological age, and self-identified race/ethnicity. Socioeconomic status was determined by the highest education level achieved, categorized into three groups: less than a high school diploma, a high school diploma or equivalent (e.g., general educational development (GED)), and education beyond college (e.g., bachelor’s, graduate, or professional degree). The study assessed clinical and anthropometric measures, including BMI as a continuous variable, and glycemic status categorized as diabetes, prediabetes, or normoglycemia. Additional health-related covariates were hypertension status (yes/no), smoking history, and patterns of alcohol consumption. NHANES laboratory assessments encompassed fasting blood glucose, HbA1c, triglycerides (TG), total cholesterol (TC), low-density lipoprotein cholesterol (LDL-C), high-density lipoprotein cholesterol (HDL-C), among other clinical indicators.

### Statistical analysis

All statistical analyses were conducted using R software, version 4.2.2, and a two-sided P value less than 0.05 was deemed significant. Based on eGDR values, participants were categorized into quartiles: quartile 1 (Q1, > 8 mg/kg/min), quartile 2 (Q2, 6 - 8 mg/kg/min), quartile 3 (Q3, 4 - 6 mg/kg/min), and quartile 4 (Q4, ≤ 4 mg/kg/min) [8].

Owing to the non-normal distribution of the data, continuous variables are summarized using the median and interquartile range (IQR). Comparisons across the eGDR quartile groups were executed using the non-parametric Kruskal-Wallis test. Univariate and multivariate logistic regression analyses were used to examine the link between eGDR and CVD. Three multivariate logistic regression models were developed to assess the independent association between eGDR and CVD, sequentially adjusting for potential confounders. Model 1 was adjusted for lifestyle and glycemic factors (glycemic status, smoking history, and alcohol use). Model 2 was further adjusted for demographic characteristics (sex, age, and race/ethnicity). Model 3 included all variables from model 2 plus additional metabolic risk factors (TC, C-reactive protein, HDL-C, triglycerides, serum creatinine, and fasting glucose levels) to evaluate the association after accounting for a comprehensive set of confounders. The effectiveness of eGDR as a continuous variable in detecting CVD was evaluated using receiver operating characteristic (ROC) curves and the associated area under the receiver operating characteristic curve (AUC). A restricted cubic spline (RCS) model was used to flexibly illustrate the dose-response relationship between log-transformed eGDR values and CVD risk. Stratified analyses were conducted to assess the consistency of the eGDR and CVD risk relationship across different predefined patient subgroups.

## Results

### Comparison of clinical characteristics across quartiles

**Figure 1:**
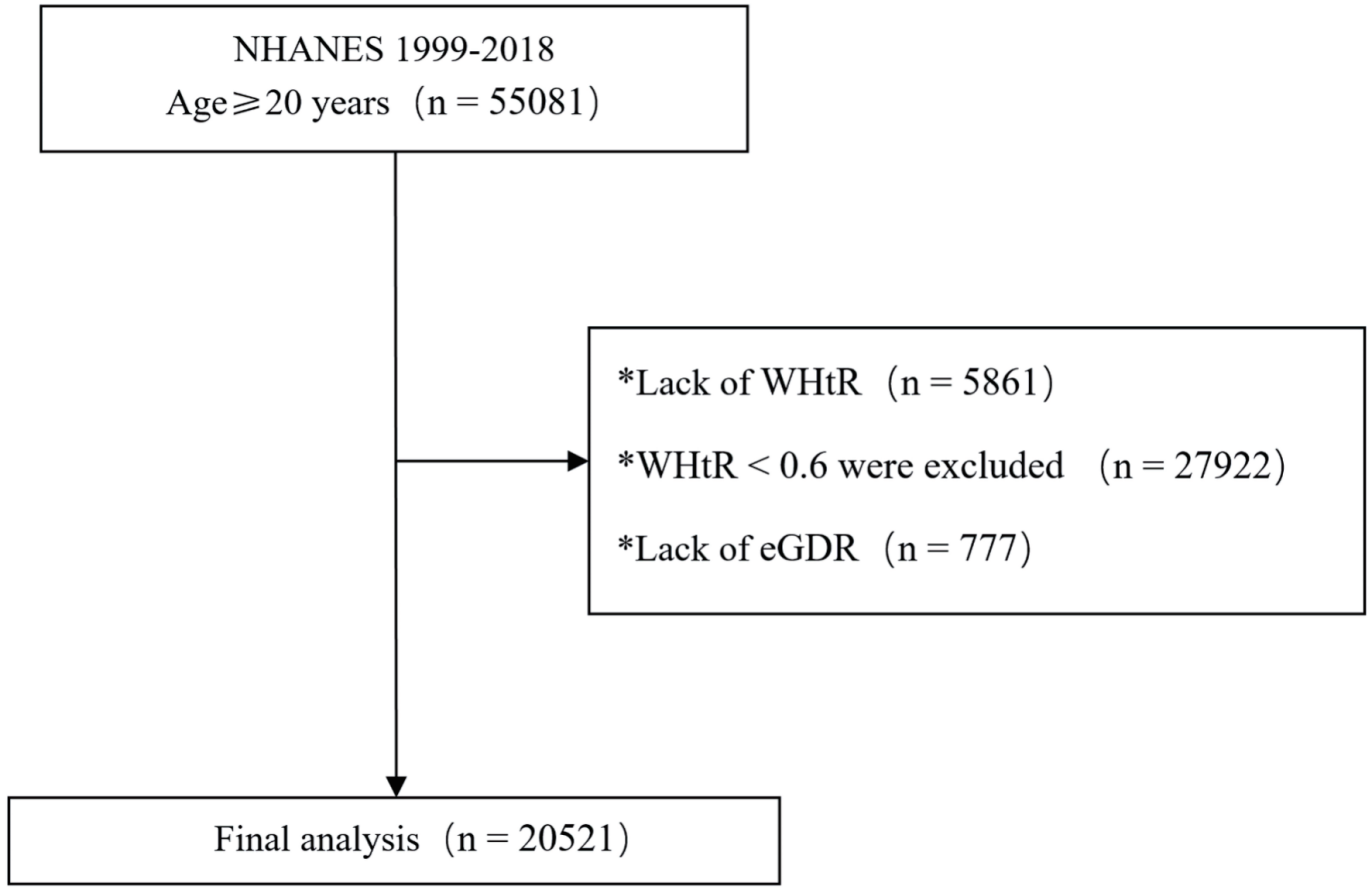
Participant flowchart. eGDR: estimated glucose disposal rate; NHANES: National Health and Nutrition Examination Survey; WHtR: waist-to-height ratio.

A total of 20,521 participants from the NHANES 1999 - 2018 cycles met the inclusion criteria (WHtR ≥ 0.6) and had complete data for eGDR and CVD outcomes, as detailed in the participant flowchart (Fig. 1). Significant differences in baseline characteristics were observed among the groups (all P < 0.001) (Table 1). The Q4 group had an average age of 58 years (± 14), with females comprising 48.4% of the group. Regarding metabolic indicators, the Q4 group had significantly higher mean BMI (39.2 ± 7.2 kg/m^2^), waist circumference (125 ± 13 cm), and HbA1c (6.98±1.79%) compared to other groups. CVD prevalence decreased with increasing eGDR. The Q4 group had significantly higher prevalence rates of CVD (26.2%), heart failure (10.2%), CAD (10.4%), and diabetes (59.6%) compared to the Q1 group (5.3%, 1.3%, 1.6%, and 5.8%, respectively). These results indicate that eGDR levels are closely associated with multiple metabolic and cardiovascular risk factors.

**Table 1:** Baseline Characteristics of Study Individuals According to Quartiles of the eGDR Index

Variables	Overall (n = 20,521)	eGDR				P value
		≤ 4 (n = 4,000)	4 - 6 (n = 6,205)	6 - 8 (n = 3,984)	> 8 (n = 6,332)	
Age (year)	54 (39, 67)	60 (49, 68)	62 (49, 72)	49 (34, 64)	44 (32, 60)	< 0.001
Female, n (%)	11,979 (58.4%)	1,936 (48.4%)	3,662 (59.0%)	2,095 (52.6%)	4,286 (67.7%)	< 0.001
Race, n (%)						< 0.001
Mexican American	4,352 (21.2%)	576 (14.4%)	1,060 (17.1%)	847 (21.3%)	1,869 (29.5%)	
Non-Hispanic Black	4,265 (20.8%)	1,217 (30.4%)	1,380 (22.2%)	762 (19.1%)	906 (14.3%)	
Non-Hispanic White	8,846 (43.1%)	1,758 (44.0%)	2,889 (46.6%)	1,807 (45.4%)	2,392 (37.8%)	
Hispanic	1,874 (9.1%)	284 (7.1%)	534 (8.6%)	343 (8.6%)	713 (11.3%)	
Other	1,184 (5.8%)	165 (4.1%)	342 (5.5%)	225 (5.6%)	452 (7.1%)	
BMI (kg/m^2^)	33.0 (30.0, 37.0)	38.1 (34.2, 42.8)	31.8 (29.4, 35.1)	35.1 (31.7, 38.7)	31.1 (29.0, 33.4)	< 0.001
WC (cm)	110 (103, 119)	124 (118, 132)	108 (103, 113)	117 (112, 122)	104 (100, 108)	< 0.001
HbA1c (%)	5.70 (5.30, 6.10)	6.40 (5.80, 7.70)	5.70 (5.40, 6.10)	5.60 (5.40, 6.00)	5.40 (5.20, 5.70)	< 0.001
Fasting glucose (mg/dL)	104 (96, 119)	122 (105, 165)	106 (98, 119)	104 (96, 115)	98 (91, 105)	< 0.001
TG (mmol/L)	1.63 (1.12, 2.41)	1.74 (1.21, 2.59)	1.64 (1.13, 2.39)	1.65 (1.14, 2.45)	1.55 (1.06, 2.29)	< 0.001
TC (mg/dL)	195 (169, 224)	185 (158, 216)	195 (169, 224)	196 (170, 223)	201 (175, 230)	< 0.001
LDL-C (mmol/L)	2.97 (2.38, 3.60)	2.74 (2.15, 3.39)	2.95 (2.33, 3.57)	3.05 (2.46, 3.65)	3.10 (2.56, 3.70)	< 0.001
HDL-C (mmol/L)	1.22 (1.01, 1.47)	1.14 (0.96, 1.37)	1.24 (1.03, 1.50)	1.16 (0.98, 1.40)	1.27 (1.06, 1.53)	< 0.001
CRP (mmol/L)	0.60 (0.25, 1.81)	0.93 (0.36, 2.96)	0.56 (0.23, 1.66)	0.64 (0.27, 2.07)	0.50 (0.21, 1.35)	< 0.001
Serum Cr (mmol/L)	72 (62, 88)	80 (66, 97)	77 (63, 91)	72 (62, 88)	67 (55, 80)	< 0.001
eGDR	6.03 (4.40, 8.31)	3.03 (2.16, 3.58)	4.95 (4.54, 5.40)	7.27 (6.65, 7.68)	8.77 (8.40, 9.19)	< 0.001
WHtR	0.66 (0.63, 0.71)	0.74 (0.69, 0.79)	0.65 (0.63, 0.69)	0.69 (0.65, 0.73)	0.63 (0.61, 0.66)	< 0.001
Hypertension, n (%)	9,782 (47.7%)	3,886 (97.2%)	5,453 (87.9%)	443 (11.1%)	0 (0.0%)	< 0.001
CVD, n (%)	2,994 (14.6%)	1,048 (26.2%)	1,256 (20.2%)	352 (8.8%)	338 (5.3%)	< 0.001
Heart failure, n (%)	942 (4.6%)	407 (10.2%)	366 (5.9%)	89 (2.2%)	80 (1.3%)	< 0.001
Coronary artery disease, n (%)	1,125 (5.5%)	412 (10.4%)	480 (7.8%)	130 (3.3%)	103 (1.6%)	< 0.001
Angina (%)	839 (4.1%)	308 (7.8%)	346 (5.6%)	89 (2.2%)	96 (1.5%)	< 0.001
Myocardial infarction, n (%)	1,177 (5.7%)	439 (11.0%)	457 (7.4%)	147 (3.7%)	134 (2.1%)	< 0.001
Stroke, n (%)	962 (4.7%)	310 (7.8%)	452 (7.3%)	98 (2.5%)	102 (1.6%)	< 0.001
Diabetes status, n (%)						< 0.001
Normal	7,609 (37.1%)	485 (12.1%)	1,875 (30.2%)	1,569 (39.4%)	3,680 (58.1%)	
Prediabetes	7,640 (37.2%)	1,132 (28.3%)	2,619 (42.2%)	1,607 (40.3%)	2,282 (36.0%)	
Diabetes	5,272 (25.7%)	2,383 (59.6%)	1,711 (27.6%)	808 (20.3%)	370 (5.8%)	
Smoking status, n (%)						< 0.001
Current	3,533 (17.2%)	675 (16.9%)	1,002 (16.2%)	751 (18.9%)	1,105 (17.5%)	
Former	5,883 (28.7%)	1,423 (35.6%)	2,024 (32.6%)	1,062 (26.7%)	1,374 (21.7%)	
Never	11,088 (54.1%)	1,901 (47.5%)	3,174 (51.2%)	2,166 (54.4%)	3,847 (60.8%)	
Alcohol consumption, n (%)						< 0.001
Heavy	759 (3.8%)	132 (3.5%)	196 (3.3%)	182 (4.7%)	249 (4.0%)	
Moderate	5,130 (25.8%)	1,074 (28.2%)	1,482 (24.8%)	1,165 (30.0%)	1,409 (22.7%)	
Mild	10,869 (54.7%)	2,043 (53.6%)	3,328 (55.6%)	1,972 (50.9%)	3,526 (56.9%)	
Never	3,121 (15.7%)	566 (14.8%)	981 (16.4%)	559 (14.4%)	1,015 (16.4%)	
NHANES cycle, n (%)						< 0.001
1999 - 2000	1,603 (7.8%)	235 (5.9%)	485 (7.8%)	295 (7.4%)	588 (9.3%)	
2001 - 2002	1,717 (8.4%)	253 (6.3%)	490 (7.9%)	338 (8.5%)	636 (10.0%)	
2003 - 2004	1,748 (8.5%)	284 (7.1%)	569 (9.2%)	327 (8.2%)	568 (9.0%)	
2005 - 2006	1,780 (8.7%)	307 (7.7%)	496 (8.0%)	337 (8.5%)	640 (10.1%)	
2007 - 2008	2,266 (11.0%)	455 (11.4%)	701 (11.3%)	427 (10.7%)	683 (10.8%)	
2009 - 2010	2,414 (11.8%)	505 (12.6%)	745 (12.0%)	493 (12.4%)	671 (10.6%)	
2011 - 2012	1,974 (9.6%)	429 (10.7%)	603 (9.7%)	395 (9.9%)	547 (8.6%)	
2013 - 2014	2,250 (11.0%)	479 (12.0%)	681 (11.0%)	424 (10.6%)	666 (10.5%)	
2015 - 2016	2,388 (11.6%)	508 (12.7%)	720 (11.6%)	469 (11.8%)	691 (10.9%)	
2017 - 2018	2,381 (11.6%)	545 (13.6%)	715 (11.5%)	479 (12.0%)	642 (10.1%)	

BMI: body mass index; WC: waist circumference; HbA1c: glycated hemoglobin; TG: triglycerides; TC: total cholesterol; LDL-C: low-density lipoprotein cholesterol; HDL-C: high-density lipoprotein cholesterol; CRP: C-reactive protein; Cr: creatinine; eGDR: estimated glucose disposal rate; WHtR: waist-to-height ratio; CVD: cardiovascular disease; NHANES: National Health and Nutrition Examination Survey.

Logistic regression analyses, both univariate and multivariate, were performed to assess the association between covariates and CVD, with CVD serving as the dependent variable (Table 2). Multivariate analysis identified the lowest eGDR quartile (eGDR ≤ 4) as an independent risk factor for CVD (P < 0.001), using the highest quartile (eGDR > 8) as the reference. All models consistently indicated a significant positive association between lower eGDR levels (Q4) and increased CVD risk (P < 0.001) (Table 3).

**Table 2:** Univariate and Multivariate Logistic Regression Analysis for CVD Risk Factors

Variables	Univariable			Multivariable		
	OR	95% CI	P value	OR	95% CI	P value
eGDR						
Q1 (> 8)	-	-		-	-	
Q2 (6 - 8)	1.72	1.47, 2.01	< 0.001	1.35	1.02, 1.78	0.035
Q3 (4 - 6)	4.50	3.97, 5.10	< 0.001	2.11	1.67, 2.66	< 0.001
Q4 (≤ 4)	6.30	5.53, 7.17	< 0.001	2.48	1.90, 3.22	< 0.001
Male	1.66	1.54, 1.80	< 0.001	1.10	0.89, 1.35	0.379
Age	1.06	1.06, 1.07	< 0.001	1.05	1.05, 1.06	< 0.001
Race						
Other	-	-		-	-	
Mexican American	0.76	0.62, 0.94	0.011	0.64	0.42, 0.98	0.039
Non-Hispanic Black	1.30	1.07, 1.59	0.010	1.01	0.67, 1.53	0.968
Non-Hispanic White	1.80	1.50, 2.18	< 0.001	1.02	0.69, 1.50	0.936
Hispanic	0.99	0.79, 1.25	0.960	0.94	0.60, 1.48	0.797
Serum Cr	1.01	1.01, 1.02	< 0.001	1.01	1.01, 1.01	< 0.001
TG	1.01	0.99, 1.03	0.280	0.91	0.56, 1.48	0.704
Fasting glucose	1.01	1.01, 1.01	< 0.001	1.00	1.00, 1.00	0.709
TC	0.99	0.99, 0.99	< 0.001	1.01	0.98, 1.03	0.711
LDL-C	0.61	0.57, 0.66	< 0.001	0.63	0.22, 1.78	0.381
HDL-C	0.70	0.63, 0.79	< 0.001	0.58	0.20, 1.66	0.307
CRP	1.01	1.00, 1.02	0.003	1.01	1.00, 1.02	0.229
Diabetes status						
Normal	-	-		-	-	
Prediabetes	1.86	1.67, 2.07	< 0.001	1.09	0.87, 1.37	0.449
Diabetes	4.41	3.97, 4.89	< 0.001	1.57	1.19, 2.06	0.001
Smoking status, n (%)						
Current	-	-		-	-	
Former	2.33	2.13, 2.54	< 0.001	1.40	1.17, 1.67	< 0.001
Never	1.46	1.31, 1.63	< 0.001	2.36	1.89, 2.94	< 0.001
Alcohol consumption, n (%)						
Never	-	-		-	-	
Mild	1.09	0.98, 1.23	0.124	1.13	0.90, 1.42	0.302
Moderate	1.06	0.93, 1.20	0.391	0.99	0.74, 1.32	0.935
Heavy	0.66	0.51, 0.86	0.002	0.82	0.51, 1.32	0.409

CVD: cardiovascular disease; TG: triglycerides; TC: total cholesterol; LDL-C: low-density lipoprotein cholesterol; HDL-C: high-density lipoprotein cholesterol; CRP: C-reactive protein; Cr: creatinine; eGDR: estimated glucose disposal rate; WHtR: waist-to-height ratio; CVD: cardiovascular disease; NHANES: National Health and Nutrition Examination Survey.

**Table 3:** Association between eGDR Quartiles and CVD Risk in Different Multivariate Models

Variables	Model 1			Model 2			Model 3		
	OR	95% CI	P value	OR	95% CI	P value	OR	95% CI	P value
eGDR									
Q1 (> 8)	-	-		-	-		-	-	
Q2 (6 - 8)	1.41	1.20, 1.66	< 0.001	1.40	1.19, 1.65	< 0.001	1.62	1.25, 2.10	< 0.001
Q3 (4 - 6)	3.37	2.95, 3.84	< 0.001	2.59	2.27, 2.95	< 0.001	3.39	2.73, 4.22	< 0.001
Q4 (≤ 4)	3.58	3.10, 4.14	< 0.001	4.10	3.57, 4.70	< 0.001	3.89	3.06, 4.94	< 0.001
Male				1.39	1.28, 1.52	< 0.001			
Age				1.06	1.06, 1.06	< 0.001			
Race									
Other				-	-				
Mexican American				0.75	0.60, 0.94	0.012			
Non-Hispanic Black				1.00	0.81, 1.24	0.994			
Non-Hispanic White				1.12	0.91, 1.36	0.291			
Hispanic				0.86	0.67, 1.10	0.219			
Serum Cr							1.01	1.01, 1.02	< 0.001
Fasting glucose							1.00	1.00, 1.00	0.917
TG							0.95	0.62, 1.45	0.806
TC							1.00	0.98, 1.03	0.694
LDL-C							0.60	0.24, 1.49	0.269
HDL-C							0.83	0.33, 2.10	0.692
CRP							1.00	0.99, 1.01	0.463
Diabetes status									
Normal	-	-							
Prediabetes	1.46	1.31, 1.64	< 0.001						
Diabetes	2.55	2.27, 2.87	< 0.001						
Smoking status, n (%)									
Current	-	-							
Former	2.06	1.87, 2.27	< 0.001						
Never	1.54	1.37, 1.74	< 0.001						
Alcohol consumption, n (%)									
Never	-	-							
Mild	0.90	0.80, 1.02	0.113						
Moderate	0.82	0.71, 0.94	0.005						
Heavy	0.58	0.44, 0.76	< 0.001						

CVD: cardiovascular disease; TG: triglycerides; TC: total cholesterol; LDL-C: low-density lipoprotein cholesterol; HDL-C: high-density lipoprotein cholesterol; CRP: C-reactive protein; Cr: creatinine; Q: quartile; eGDR: estimated glucose disposal rate; OR: odds ratio; CI: confidence interval.

### Association of eGDR with different CVDs

**Figure 2:**
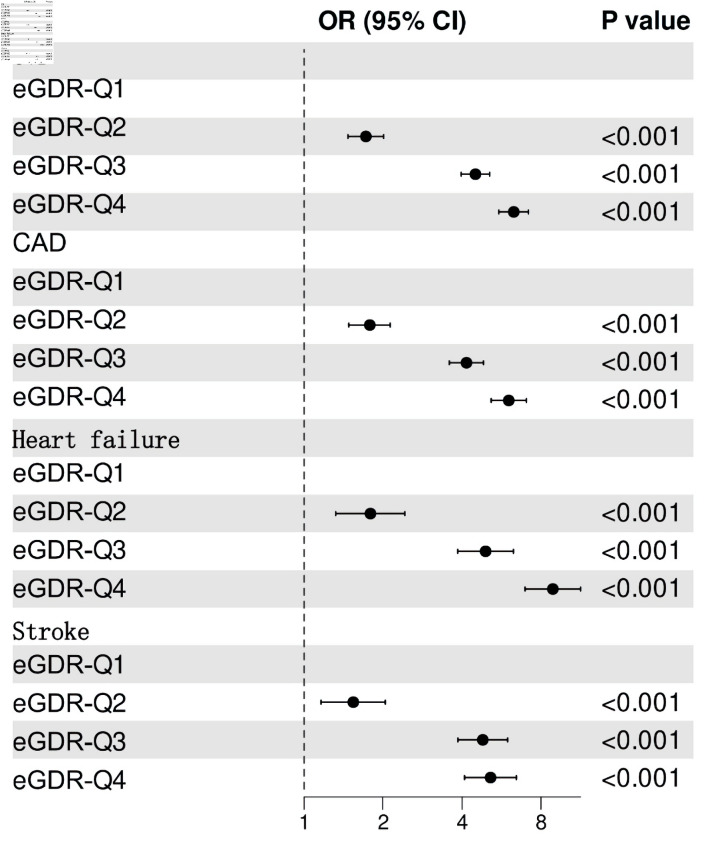
Adjusted odds ratios (ORs) for cardiovascular diseases (CVD) by eGDR quartile (Q4 vs. Q1). eGDR: estimated glucose disposal rate; CI: confidence interval; Q: quartile; CAD: coronary artery disease.

**Figure 3:**
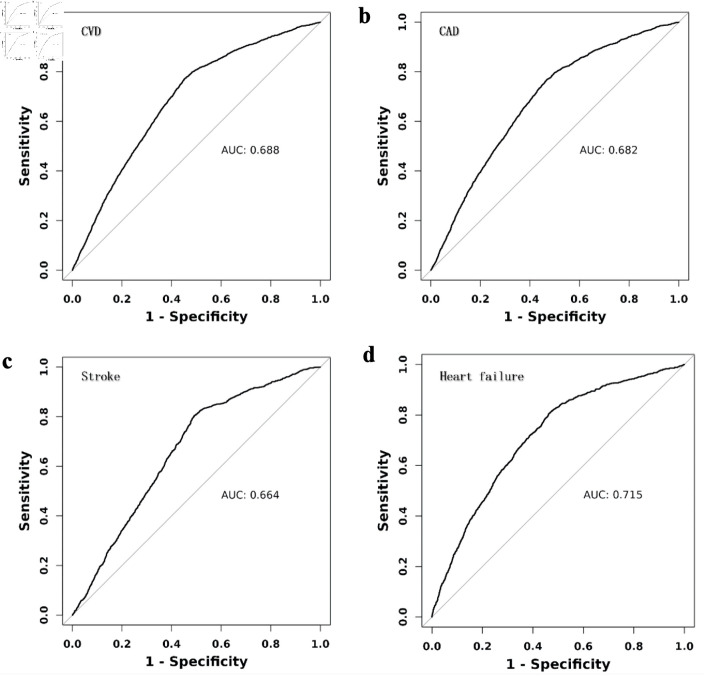
Receiver operating characteristic (ROC) curves for eGDR in predicting cardiovascular diseases (CVD). CAD: coronary artery disease; AUC: area under the receiver operating characteristic curve.

**Figure 4:**
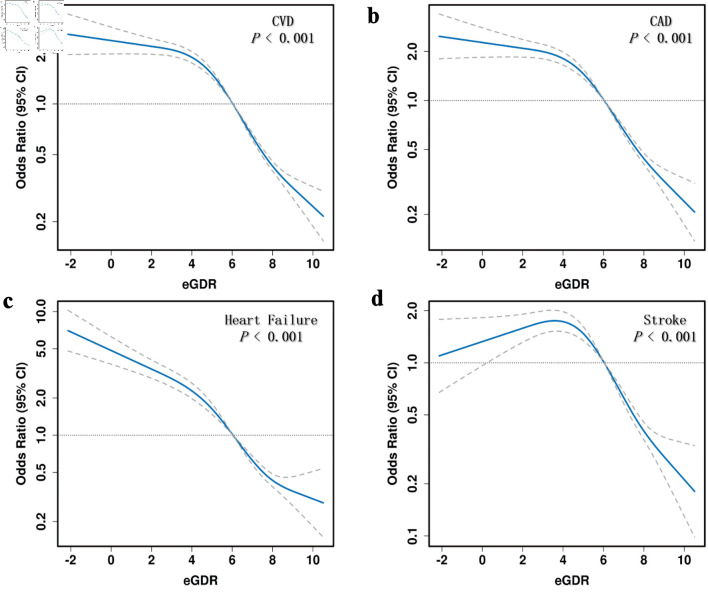
Restricted cubic spline (RCS) analysis of the association between eGDR and cardiovascular disease (CVD) risk. eGDR: estimated glucose disposal rate; CAD: coronary artery disease; CI: confidence interval.

Figure 2 illustrates that participants in the lowest eGDR quartile (Q4) had significantly higher adjusted odds ratios (ORs) for CVD (OR = 6.3, 95% confidence interval (CI): 5.53 - 7.17), CAD (OR = 6.03, 95% CI: 5.17 - 7.04), heart failure (OR = 8.88, 95% CI: 6.96 - 11.33), and stroke (OR = 5.14, 95% CI: 4.09 - 6.45) compared to those in the highest quartile (Q1). ROC analysis demonstrated that eGDR achieved the highest AUC values for predicting CVD (0.688, 95% CI: 0.678 - 0.698), CAD (0.682, 95% CI: 0.670 -0 .693), heart failure (0.715, 95% CI: 0.699 - 0.730), and stroke (0.664, 95% CI: 0.648 - 0.679) (Fig. 3). Figure 4 illustrates that the RCS logistic regression models showed a strong inverse link between eGDR and the risk of CVD and its subtypes.

### Analysis of the correlation between eGDR and CVD across different subgroups

The analyses of subgroups indicated that the strong connection between reduced eGDR levels (Q4) and elevated CVD risk was consistent across various age categories (≤ 60 vs. > 60 years), sexes (male vs. female), and glycemic statuses (non-diabetes, prediabetes, diabetes), with all P values < 0.001 (Table 4).

**Table 4:** Subgroup Analysis of the Association Between eGDR Quartiles (Q4) and CVD Risk

Variables	Age		Sex		Diabetes status		
	≤ 60	> 60	Male	Female	Normal	Prediabetes	Diabetes
eGDR Q1	-	-	-	-	-	-	-
eGDR Q2	1.79 (1.35 - 2.36)	1.40 (1.15 - 1.71)	1.16 (0.93 - 1.46)	2.19 (1.77 - 2.72)	1.34 (1.02 - 1.77)	1.49 (1.16 - 1.92)	1.04 (0.74 - 1.45)
	P < 0.001	P < 0.001	P = 0.182	P < 0.001	P = 0.036	P = 0.002	P = 0.833
eGDR Q3	5.73 (4.56 - 7.20)	2.17 (1.86 - 2.55)	3.60 (2.99 - 4.33)	5.09 (4.28 - 6.05)	4.40 (3.57 - 5.41)	3.92 (3.21 - 4.80)	1.91 (1.42 - 2.56)
	P < 0.001	P < 0.001	P < 0.001	P < 0.001	P < 0.001	P < 0.001	P < 0.001
eGDR Q4	8.84 (7.03 - 11.12)	3.23 (2.73 - 3.81)	5.04 (4.19 - 6.07)	6.53 (5.42 - 7.86)	3.26 (2.37 - 4.50)	4.51 (3.60 - 5.66)	2.35 (1.76 - 3.15)
	P < 0.001	P < 0.001	P < 0.001	P < 0.001	P < 0.001	P < 0.001	P < 0.001

Q: quartile; eGDR: estimated glucose disposal rate.

## Discussion

This research uniquely validates the predictive value of eGDR in individuals with central obesity (WHtR ≥ 0.6). A significant inverse relationship between eGDR and CVD risk was observed after controlling for potential confounders. Individuals with the lowest eGDR levels (≤ 4 mg/kg/min) had a 6.3-fold increased risk of CVD (95% CI: 5.53 - 7.17). RCS analysis revealed a nonlinear, inverse dose-response relationship between eGDR and CVD, including its subtypes. Subgroup analyses based on various demographic and clinical characteristics further confirmed the robustness of our findings. The findings indicate that eGDR may be useful for early detection and screening of individuals at high risk for CVD, highlighting the significance of weight control and reducing IR to lower CVD risk.

Since its development, the eGDR has been widely used in studies assessing chronic complications in diabetic populations [3]. A study involving 2,151 individuals with type 1 diabetes (T1D) found a strong association between eGDR and both microvascular and macrovascular complications [9]. According to Zabala et al, a higher eGDR was correlated with decreased chances of stroke (hazard ratio (HR) = 0.60; 95% CI: 0.48 - 0.76) and mortality (HR = 0.60; 95% CI: 0.48 - 0.76) among patients suffering from type 2 diabetes [10]. A study based on NHANES data found a linear correlation between reduced eGDR and a higher occurrence of CVD in individuals with prediabetes [11]. eGDR is pertinent for complications in diabetic patients and also holds predictive significance in non-diabetic populations. Huang et al [12] found that each one-unit rise in eGDR was associated with reduced risks of various cardiovascular conditions: 12% for myocardial infarction, 20% for heart failure, 15% for atrial fibrillation, and 13% for ischemic stroke in the general population. Yi et al [13] found that a 17% decrease in the risk of atherosclerotic CVD in the general population was correlated with a 1-standard deviation (SD) increase in eGDR. According to the CHARLS study, non-diabetic people experienced a 13% lower risk of heart disease and a 30% lower risk of stroke with every 1-SD increase in eGDR [14]. The research shows that reduced eGDR levels are negatively correlated with CVD, stroke, and heart incidents, emphasizing its importance for CVD screening in obese groups. In addition to eGDR, indices like homeostasis model assessment of insulin resistance (HOMA-IR) and metabolic score for insulin resistance (METS-IR) that measure IR are associated with CVD risk. High HOMA-IR levels independently predict major adverse cardiovascular events and all-cause mortality, with a notably increased risk in patients exhibiting elevated HOMA-IR [15]. In contrast, the METS-IR index exhibits a non-linear, U-shaped correlation with CVD mortality, suggesting its potential utility as a predictive biomarker for assessing mortality risk among patients with established CVD [16].

The causal relationship between IR and CVD remains a focus of research and debate. IR often accompanies various CVD risk factors, including obesity, hypertension, and dyslipidemia, complicating the assessment of whether it directly contributes to CVD or influences it indirectly via other mediators. Mendelian randomization (MR) analysis using 53 single-nucleotide polymorphisms (SNPs) linked to IR phenotypes as instrumental variables revealed that a genetically forecasted 1-SD elevation in IR was connected to greater hypertension risks (OR = 1.06, P < 0.001), peripheral artery disease (OR = 1.90, P < 0.001), and heart failure (OR = 1.19, P = 0.041), suggesting IR may causally contribute to CVD [17]. Another MR analysis by Huang et al [12] established a causal link between visceral adipose tissue (VAT) mass and both IR and various CVD endpoints. There was an association between genetically predicted larger VAT mass and elevated IR (OR = 1.204, 95% CI: 1.16 - 1.25), and significant associations with coronary heart disease, myocardial infarction, and heart failure, indicating that visceral fat accumulation elevates CVD risk by worsening IR [18]. The strong inverse association between eGDR and CVD risk likely stems from the central role of IR in promoting atherosclerosis through multiple pathways [19]. These include chronic inflammation, vascular endothelial dysfunction, and dysregulated lipid metabolism. Dysregulated lipid metabolism, often seen in obesity through elevated triglycerides, increased small dense LDL particles, and intracellular lipid buildup, is strongly linked to IR, heightening the risk of type 2 diabetes and CVD [20]. This suggests that IR indirectly contributes to CVD risk via its effects on lipid metabolism [21, 22]. While other indices, such as HOMA-IR, are valuable, eGDR offers a distinct advantage in obesity-related CVD risk assessment. Its formula incorporates waist circumference, a direct measure of central adiposity, and hypertension, a key cardiometabolic comorbidity [23]. This makes eGDR a more holistic surrogate that captures both the metabolic and hemodynamic manifestations of IR, which is particularly relevant in an obese population.

As a convenient assessment tool, and based on the high-risk threshold of eGDR ≤ 4 mg/kg/min (which is associated with a 26.2% CVD prevalence in Q4), we propose incorporating eGDR into routine evaluation in obesity clinics: 1) Prioritize individuals with eGDR ≤ 4 for advanced screening (e.g., echocardiography or coronary computed tomography angiography (CTA)); 2) Use eGDR as a dynamic monitoring indicator to assess the efficacy of lifestyle or pharmacological interventions (for example, an increase in eGDR ≥ 1 unit post-intervention may indicate reduced risk). This method offers a valuable clinical tool for early detection of individuals at elevated CVD risk, enabling prompt and specific interventions.

### Study limitations

This study is a cross-sectional analysis based on a US adult population database, and its applicability to other demographic groups requires additional empirical validation. While a multitude of statistical models were employed to adjust for potential confounding variables, the possibility of residual confounding cannot be entirely discounted, primarily attributable to the self-reported, questionnaire-based methodology of certain data sources.

### Conclusions

In summary, our study highlights the significant association between lower eGDR levels and increased CVD risk in individuals with obesity as defined by WHtR ≥ 0.6. We found that individuals affected by obesity with low eGDR levels exhibit a substantially elevated risk of composite CVD events. Examining the dose-response relationship between temporal changes in eGDR (ΔeGDR) and CVD events can offer important insights for identifying the best timing for interventions.

## Data Availability

This publication includes the data and original contributions that support the study’s findings. Additional information can be obtained from the corresponding authors upon reasonable request.
